# CONTACT: AN ONLINE COACHING TOOL TO EMPOWER CARE PARTNERS TO FACILITATE CONVERSATIONS IN NURSING HOMES

**DOI:** 10.1093/geroni/igad104.2834

**Published:** 2023-12-21

**Authors:** Gail Towsley, Alexandra Terrill, Gregory Bayles

**Affiliations:** University of Utah, Salt Lake City, Utah, United States; University of Utah, Salt Lake City, Utah, United States; University of Utah, Salt Lake City, Utah, United States

## Abstract

In the post COVID-19 context, nursing homes struggle with staffing. The role of care partners (CPs; relative, friend) has never been more crucial, including the need to advocate for persons living with dementia (PLWD) and being an integral part of the care team. The online CONversaTion cAre Coaching Tool (CONTACT) empowers CPs of PLWD prepare for conversations that may include communicating what matters to the PLWD, need for a different type of care, or treatment decisions. We engaged CPs to complete and provide feedback on CONTACT, which includes four sections: 1) education about nursing homes to clarify key terms/processes 2) guidance on goal setting to identify what is important; 3) creation of action plan to achieve the goal; and 4) reflection on the impact of achieving the goal. CPs (n=8) completed a survey about using the tool (acceptability (4 items), appropriateness (4 items), feasibility (4 items); scale of 1=strongly disagree to 5=strongly agree), and participated in a focus group to discuss the tool’s content and function. CPs favorably rated the tool. Mean ratings for acceptability ranged from 4.13 – 4.25, appropriateness 3.88 – 4.43, and feasibility 4.13 – 4.57. CPs reported that the tool was clear, simple, logically organized, informative, and it provided direction for starting a conversation. They liked the definitions of terms, large response buttons and that instructions were provided via video and in writing. Recommendations included additional videos depicting conversation prompts and scenarios, and utilizing community-based programs such as the Ombudsman office to disseminate CONTACT.

